# Unlocking employee innovative behavior: the role of humble leadership, core self-evaluation, and leader-member exchange

**DOI:** 10.1186/s40359-024-01668-y

**Published:** 2024-03-26

**Authors:** Gaofeng Wang, Laiba Saher, Tang Hao, Asad Ali, Muhammad Waqas Amin

**Affiliations:** 1 School of Public Administration and Sociology, Jiangsu Normal University, Xuzhou, Jiangsu China; 2 University of the Punjab, Lahore, Pakistan; 3 School of business administration, ZhongNan University of Economics and Law, 430073 Wuhan, China; 4 Foundation University, Islamabad, Pakistan; 5 School of Management, Yanshan University, Qinhuangdao, China

**Keywords:** Humble leadership, Leader-member exchange, Core self-evaluation, Innovative behavior

## Abstract

Humble leadership has gained attention in recent years due to its potential impact on employee performance. This study explores the association between humble leadership and follower innovative behavior by investigating the moderating role of core self-evaluation (CSE) and the mediating role of leader-member exchange (LMX). The study uses data from 328 followers and their immediate leaders to test a mediated moderation model. Results show that there is a favorable association between humble leadership and LMX and followers’ innovative behavior, particularly pronounced for followers who possess lower levels of CSE. The findings suggest that humble leaders should focus their development efforts on followers with low CSE to achieve complementarity congruity and improved innovation. This research enhances the existing body of knowledge by emphasizing the significance of comprehending the functions of relational procedures and the psychological resources of followers in determining the effectiveness of humble leadership. These findings have practical implications for organizations seeking to enhance their leadership effectiveness and followers’ innovative behavior.

## Introduction

In today’s dynamic workplace, humble leadership, which is a value-based approach, can serve as a crucial element in promoting positive behavior among followers [1, 2]. Humble leadership is defined as a combination of values, attitudes, and behaviors exhibited by the leader that is characterized by accurate self-appraisal, a recognition of others’ strengths, an appreciation for contributions, and a willingness to accept criticism and ideas from others [3]. Humble leaders are distinguished by their capacity to foster well-being, develop follower’s confidence in their abilities to achieve goals. Humble leaders emphasize transparency, and ethical behavior, prioritize the personal growth and well-being of their followers, promote a sense of shared community and purpose, and incorporate humble values and beliefs into their leadership approach [4]. Such an approach facilitates followers’ accurate self-awareness, and understanding of humble leadership actions [5]. Scholarly evidence indicates humble leadership stye is practiced by leaders in a variety of organizational settings such as, healthcare [6], banking and finance [7], education [8], manufacturing [9], and hospitality [10]. Despite the value that humble leadership brings to both followers and organizations, research on the mechanisms and processes through which humble leadership influences employees’ innovative behavior is still in its infancy [2, 11]. This study addresses this limitation by exploring processes and conditions that explain how humble leadership contributes to improving followers’ innovative behavior.

The theory building about mechanism and processes of humble leadership are still developing [2, 11]. Efforts have been dedicated to understanding the developmental processes that underlie the relationship between humble leadership and the attitudes and behaviors of followers [2, 6, 12]. For an instance, Owens and Hekman [13] proposed a humble leadership model based on three components: self-appraisal, recognition of others, and openness to criticism and experience. Other researchers have drawn on concepts such as mindfulness, compassion, and wisdom to describe the underlying mechanisms of humble leadership [8, 12–14]. Overall, the body of research indicates that humble leadership has the capacity to inspire and shape follower effectiveness, yet there remains a need for a more nuanced comprehension of the individual and situational variables that could mediate and moderate the influence of humble leadership on the innovative behavior of subordinates. More specifically, a recent meta research [15] has suggested to shed light on followers’ persona and contextual factors that may influence how humble leadership shapes followers’ behavior and performance.

An argument posits that humble leaders can cultivate and sway their followers by stimulating them with constructive mental states that promote their capabilities [16]. Given that employees may vary in their susceptibility to such influence, it remains unclear whether humble leadership can consistently affect their performance. Such questioning is rooted in the complementary congruity perspective [17, 18]. According to the theory, the leader’s strengths can complement the weaknesses of the follower and help them in fulfilling their goals.

Based on the complementary congruity theory [17, 18], this study suggests that the humble leader can play a vital role in enhancing the follower’s innovative behavior by providing complementary capabilities. Follower innovative behavior is defined as a process through which employees recognize problems, generate ideas, and find resources to implement ideas in order to solve the problems [19]. Innovative behavior is critical for employee development [20], survival and growth of organization and contributes to gain competitive advantage in global competitive business environment [21]. Considering the importance of innovative behavior for individuals and organization, it is important to investigate the ways to improve employee innovative behavior. In this regard, research has suggested that leadership plays critical role in improve followers’ innovative behavior [22]. As such, humble leadership, a value-based leadership, is proposed to influence follower innovative behavior [23]. The humble leader’s attributes complement the missing or required abilities of the followers, contributing to their overall effectiveness through exchange relationships with followers. Fry, Latham [24] proposed that humble leaders utilize their reserves of positive self-esteem to boost the core capabilities and self-evaluation of their followers, thereby improving their innovative behavior. This concept has led to the development of CSE [25, 26], a widely recognized construct that encompasses hope, transcendence, self-efficacy, and optimism as self-esteem resources.

Based on the premise of individual variations among followers and utilizing the theoretical framework of complementary congruity, our primary aim is to investigate whether the association between humble leadership and the performance of followers is contingent on the extent of the follower CSE. Additionally, we endeavor to scrutinize a potential mechanism that could clarify the conditional impact of CSE. Specifically, our investigation centers around the relational dynamics of leader-member exchange (LMX) as an intermediate mechanism that links humble leadership with the innovative behavior of followers.

Our study aims to scrutinize the process of LMX as the underlying mechanism by which humble leaders impact the innovative behavior of followers due to two reasons. Firstly, leadership is widely recognized as a relational process [27] where the nature and quality of the leader-follower relationship plays a crucial role in shaping follower responses to leader behaviors [28]. Secondly, to fully comprehend the contingent weight of CSE on the humble leadership-follower innovative behavior association, we must investigate the process that is most relevant to the complementary congruity mechanism. Previous studies have demonstrated that leaders can cultivate favorable psychological states in their subordinates through ongoing interaction and meaningful exchange relationships [29]. As such, we suggest that the followers of humble leaders are offered complementary congruity with the consequential creative behavior impact. However, the question remains unanswered as to if subordinates with varied levels of CSE may reap greater or lesser benefits from their exchange relationship with the leader. The phenomenon of varying stages of adherents’ CSE in the LMX might account for the variable impact of humble leadership on adherent innovative behavior. Hence, we are not only concerned to explore if CSE moderates the relationship between LMX and follower innovative behavior relationship, but also whether LMX serves as a mediator between humble leadership and follower innovative behavior.

**Fig. 1:**
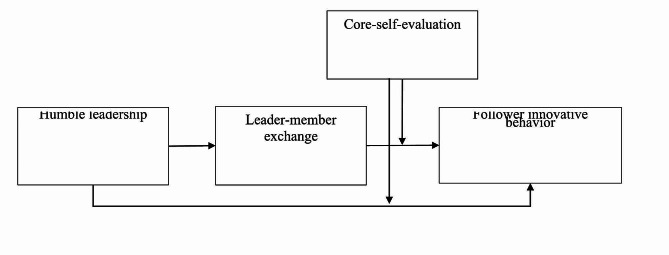
Theoretical model

In essence, theoretical model of this study, presented in Fig. 1, aims to provide significant contributions to both humble leadership and CSE by presenting a well-rounded and inclusive viewpoint that acknowledges the pivotal part played by followers’ positive psychological resources in determining the effectiveness of humble leadership. To this end, we adopt the theoretical framework of complementary congruity, which emphasizes the importance of understanding the congruence between leader and follower in terms of their respective psychological resources. By investigating how humble leadership enhances follower innovative behavior through LMX relationships while accounting for the moderating impact of followers’ CSE, our study highlights the importance of combining both the psychological resources of followers and relational dynamics into a unified framework for assessing the effectiveness of humble leadership.

## Theory and hypotheses

Complementary congruity theory suggests that individual capabilities complement the capabilities of others to satisfy their tasks requirements [17, 18]. In other words, complementary congruity theory suggests that leadership behavior plays a critical role in shaping how employes perform their tasks [30]. For instance, humble leadership behavior influences the motivation and confidence of their followers in their capabilities to perform their job-related tasks. As a result, humble leadership improves followers’ innovative behavior.

Building upon the theoretical foundation described, the three dimensions of humble leadership - a) a willingness to accurate self-appraisal, (b) a recognition of others’ strengths and contributions, and (c) acceptance of criticism and openness to new ideas - offer a holistic perspective on effective leadership [3, 31]. Firstly, it highlights the importance of embracing an honest and precise self-perception, allowing leaders to acknowledge their own strengths and weaknesses. Secondly, the article underscores the significance of recognizing and valuing the strengths and contributions of others within the team, fostering a collaborative and supportive environment. Lastly, the concept of teachability emerges as a crucial trait, emphasizing the leader’s openness to novel ideas and constructive feedback. By encompassing these elements, humble leadership provide an integrated and comprehensive approach to understanding and measuring the effectiveness of leadership in a humble context.

### Humble leadership and employee innovative behavior

We anticipate that humble leadership will exert a favorable impact on the innovative behavior of followers [32]. Past theoretical analyses suggest that humble leaders have the capacity to shape the innovative tendencies of their subordinates [33]. Humble leaders embody their values and endeavor to attain candor and veracity in their interactions with their followers [4]. Humble leaders can lead through exemplary behavior, setting the tone where trust and honesty are encouraged [34]. Exemplifying leadership entails displaying unwavering devotion to one’s responsibilities and serves as a beacon of guidance to followers regarding ways to maintain cognitive alertness and emotional and physical engagement during work. Zhou and Wu [33] suggests that humble leaders’ ethical behavior can serve as a model for their followers due to their integrity and appeal as role models.

Further, humble leadership followers tend to ascribe leaders with strong positive qualities, adopt their values and beliefs, and exhibit consistent behavior in alignment with them. As posited by Caldwell, Ichiho [35] the conduct of humble leaders is perceived by their adherents to be guided by elevated ethical principles and distinguished by impartiality, candor, and rectitude in their interactions with their followers. Henceforth, these leaders can foster shared values among their subordinates through their exemplification of transparency, positivity, and a strong ethical code. As a consequence, followers are incentivized to demonstrate constructive conduct and experience a feeling of self-esteem and a responsibility to respond in kind [36, 37].

Furthermore, in addition to the theoretical foundations that explain the positive influence of humble leaders on their followers’ creativity, there is also growing empirical evidence to support this claim. For instance, research by Rego, Owens [5] found a positive correlation between humble leadership behavior and the job performance through enhancing psychological empowerment of team. Additionally, Tariq, Abrar [14] noted that humble leaders inspire followers by demonstrating and instilling a strong nous of concern for achieving progressive results in service performance. Based on the insights obtained from the literature, we formulate a hypothesis for our study:

*H1: Humble leadership is positively related to employee innovative behavior.*

### The moderating role of core self-evaluation

As the complementary congruity theory postulates that the extent to which leaders’ behaviors or competencies align with the corresponding needs of their followers is a crucial determinant of effective leadership [17]. We contend that in situations where there is a lack of complementarity between a leader’s competencies and the characteristics of their followers, the leader’s influence may be diminished. This is since the need for the leader’s influence is greatly reduced. We contend that in situations without complementarity between the capabilities of leaders and the characteristics of their followers, leaders may have limited influence since the need for follower development is reduced [38]. Conversely, when a leader’s specific competencies complement the necessities of their followers, they are likely to significantly enhance their followers’ performance in a particular area. Building on the complementarity perspective, we suggest that while humble leadership can boost the innovative behavior of followers who require positive psychological resources, this benefit may strengthen when they possess a high level of CSE, indicating they already possess self-esteem, locus of control, neuroticism, and self-efficacy.

Humble leadership and CSE have comparable effects on enhancing follower job performance through the cultivation of positive psychological resources. The four elements of CSE, including self-esteem, locus of control, neuroticism, and self-efficacy, are deemed vital psychological resources that can contribute to favorable organizational outcomes [39]. According to Booth, Shantz [40], CSE demonstrates a notable effect on favorable employee attitudes, behaviors, and outcomes assessed through several measures. As initially illustrated by Fry [41], the behaviors of humble leaders originate from these constructive psychological resources and subsequently result in the growth of both themselves and their followers [25, 42].

It is suggested that humble leadership leads to positive outcomes for followers because it fosters positive CSE [43]. In particular, humble leaders possess the capacity to maintain a realistic sense of hope and integrity and can augment their followers’ sense of hope by not only strengthening their motivation but also by emphasizing positive courses of action, which can boost their followers’ sense of self-efficacy [44]. Furthermore, humble leaders possess the ability to construe information, exchanges, and collaborations with followers through a progressive lens. Resultantly, they tend to have the capacity to evoke positive emotions amongst their adherents, ultimately leading to the development of a sense of optimism [16, 41, 45]. Research findings have demonstrated that humble leadership is positively linked to both leaders’ and followers’ CSE, which ultimately results in improved innovative behavior among followers [46, 47]. However, it remains to be examined whether CSE can moderate the relationship between humble leadership and follower innovative behavior.

The positive influence of humble leadership on followers’ innovative behavior can be explained by the complementarity perspective. The complementary congruity process suggests that the positive influence of humble leaders is more pronounced when followers possess CSE states, though the influence inclines to diminish where the followers lack adequate CSE levels. In other words, individuals with high CSE possess positive psychological resources, such as self-esteem, locus of control, neuroticism, and self-efficacy, that inspire them to attain high levels of performance. In such a situation, followers also understand their resource needs and lack of psychological abilities. Conversely, high CSE individuals are more willing to attain positive development and behaviors instilled by humble leaders to enhance their performance [38]. Therefore, humble leaders’ positive behaviors and development supplement the psychological resource of high CSE followers, leading to raised innovative behavior. Based on the provided context, the study hypothesis can be derived as follows:

*H2: Followers’ CSE moderates the relationship between humble leadership and innovative behavior of followers, such that the relationship is stronger among followers with high rather than low levels of CSE.*

### The mediating role of leader-member exchange

Having established with evidence from the literature the role of CSE as a moderator for the relationship between humble leadership and follower innovativeness, we can now examine the potential mediating process that may account for the overall moderated effect of humble leadership. Based on our initial discussion regarding relational processes, we anticipate that LMX will serve as a mediator in the association between humble leadership and the innovative behavior of followers. In particular, we highlighted that humble leadership represents a genuine and collaborative connection that is established amongst a leader and their followers. This dynamic and genuine bond can foster favorable social interactions by establishing integrity and earning trust and respect among followers [48, 49]. The relationships formed through these exchanges have been found to lead to successful followers’ innovative behavior.

Humble leadership has the potential to impact the creation and preservation of reciprocal relationships with followers [50]. The constituents of interconnectedness, the search for purpose, meaning, and connection among individuals’ relationships collectively manifest the vision, hope/faith, and altruistic love of humble leaders [13]. These traits represent the fundamental components of exchange relationships of superior quality. Firstly, humble leaders possess the ability to solicit varied perspectives from their followers, demonstrating an appreciation for and reliance on their input [6]. This action is expected to be encountered with reciprocal trust and respect from the adherents toward the leader. Secondly, humble leaders exhibit faith and strong moral principles, which allows them to be perceived by their followers as honest and trustworthy [51]. Consequently, their followers are more likely to cooperate with them and have greater trust in their leadership [48, 52]. Thirdly, humble leaders exhibit transparency by openly conveying their qualities, values, goals, and weaknesses to their followers, while motivating them to reciprocate similar behavior. This approach fosters faith and affection between the leader and followers, leading to an open and productive exchange of ideas [33]. Furthermore, it should be noted that relational transparency not only involves open communication but also entails a sense of accountability on the part of leaders [28, 53]. Fostering accountability within relationships with followers involves creating a mutual understanding of forthcoming actions and individual responsibilities, which can ultimately result in the formation of strong, high-quality relationships built over time [28]. On basis of this discussion, we hypothesis as:

*H3: humble leadership is positively related to followers’ LMX.*

In addition to the connection between humble leaders and their followers’ LMX, the assumption that followers should respond with innovative behavior in exchange for the treatment they receive from the leader forms the basis for the positive association between LMX and follower innovative behavior [54–56]. To be more specific, when the quality of LMX is low, it typically leads to that is standard or regular innovative behavior since the exchanges within these associations are transactional and primarily based on fulfilling contractual obligations [54]. High-quality LMX, on the other hand, results in exceptional innovative behavior due to the transformation of the relationship from purely an economic exchange to a socially oriented exchange marked by reciprocal respect, trust, and obligation [28]. Over time, a considerable body of empirical evidence has consistently shown a positive correlation between LMX and diverse work outcomes for subordinates [27, 29]. To summarize, LMX can be considered a predictor of followers’ innovative behavior. In light of these findings, we propose a hypothesis for our study as:

*H4: LMX mediates the relationship between humble leadership and follower innovative behavior.*

### The mediated moderation relationship

Despite a well-established positive association between LMX and subordinate creativeness, researchers in this area have continuously advocated for the investigation of moderators, especially individual differences that may impact the relationship between LMX and innovative behavior [57, 58]. Scholars have proposed that although a high-quality LMX can be beneficial in providing followers with guidance, resources, and support, its influence may be constrained when alternative sources of support and motivation are accessible to followers [59]. While we concur with this perspective, we would also propose that individuals who possess low levels of CSE might not fully leverage the advantages of their high-quality LMX relationships with their leader compared to those with high levels of CSE. Therefore, the association between LMX and followers’ innovative behavior is anticipated to fluctuate depending on the followers’ level of CSE.

Previous research by Bauer, Erdogan [59] suggests that the LMX-follower positive correlation and follower innovative behavior is, to some extent, attributable to the tangible and intangible rewards that high-quality LMX can offer to followers. Among those rewards are the actions exhibited by leaders, such as providing followers with job feedback information [28], shielding followers from negative consequences, and facilitating the acquisition of resources [60]. Additional advantages of high-quality LMX for followers include being introduced to valuable social networks, receiving favorable job assignments [61], protection against unfair treatment, encouragement to take on challenging tasks, and provision of emotional support and affection [28, 61]. Put simply, leaders can create positive or negative conditions for their followers’ performance by cultivating high or low-quality relationships with them [57], which in turn can affect their physical and psychological well-being and subsequent level of outcomes.

As previously mentioned, CSE refers to a collection of constructive mental assets that enhance an individual’s drive to achieve objectives and targets [62]. Empirical research has established that CSE has a positive influence on followers’ innovative behavior, both subjectively and objectively, in both experimental and longitudinal studies [25, 43, 63]. The aforementioned findings imply that the aid and resources provided through high-quality LMX may become more vital. Hence, followers with high CSE are likely to take LMX relationships to facilitate their innovative behavior. Conversely, low CSE followers may struggle to persevere through challenging circumstances, sustain an optimistic perspective, and remain less motivated to pursue success without the assistance and resources derived from a high-LMX relationship. Resultantly, followers with high CSE could rely more on LMX for innovative behavior compared to those with low CSE. In short, high CSE followers are more likely to seek benefits from LMX to demonstrate innovativeness. Therefore, LMX is expected to have a greater effect on the performance of low CSE followers than high CSE followers. Therefore, we draw the following hypothesis:

*H5a: CSE moderates the relationship between LMX and follower innovative behavior, such that the relationship between LMX and follower innovative behavior is stronger among followers with high rather than low levels of CSE.*

Particularly, humble leadership impact on follower innovative behavior is moderated by CSE, as a result of the mediating role of the quality of the leader-member exchange on the relationship between humble leadership and follower innovative behavior, and the moderating role of the follower’s CSE on the relationship between the quality of the leader-member exchange and follower innovative behavior. Furthermore, high-level CSE followers are more likely to benefit from the supportive behaviors of humble leaders and the resulting high-quality LMX, leading to greater levels of innovative behavior compared to low-CSE followers. Therefore, our study proposes that the relationship between humble leadership, LMX, and innovative behavior is more solid for high CSE followers compared to low CSE followers. In light of this discussion, we propose a hypothesis for our study:

*H5b: The mediation of LMX underlies the overall moderating effect of CSE on the relationship between humble leadership and follower innovative behavior in such a way that humble leadership is positively related to LMX, and the relationship between LMX and follower innovative behavior is stronger among followers with high rather than low levels of CSE.*

## Method

### Sample and procedure

To test the model of this study, a total of 328 frontline service employees and their 45 supervisors participated in three questionnaire surveys. Hotels play a critical role in the growth of the service industry in the economies of emerging countries such as China [64–66]. Although, creativity and innovation may not be the core job of frontline service employees, however, in the hospitality industry, management encourages generating an idea and implementing new and useful approaches for efficient service leading to customer satisfaction. Therefore, the sample from hotels provides useful content to test the model of this study. Authors used online booking app to identify hotels rated four-star by customers. Initially, the authors contact the HR managers of these six hotels, and four hotels agreed to take part in the survey.

We conveyed that participation is voluntary and assured HR managers that the data collected from the employees will not be shared with anyone and will be used for this study only. With the assistance of HR departments of sample organizations, questionnaires were distributed to 500 frontline service employees in the first phase. In this first phase survey, employees were asked to report demographic characteristics and humble leadership, and employee CSE. In the first phase survey, we received 380 complete responses. A month later in the second phase survey were asked to report leader-member exchange and we received 349 responses. One month after the second phase survey, we distributed questionnaires to 59 leaders and asked them to report the innovative performance of their subordinates. As a result, we receive 335 responses from leaders. After matching the first, second, and third phase response, we found 331 matched responses from employees. After removing 3 incomplete responses, we have 328 useful responses (response rate = 66%).

We employed an analysis of nonresponse bias using the approach recommended by previous research [67]. We conducted a chi-square test on the demographic variables of the first 25% and the final 25% of the sample. The results found no significant difference between the two groups, suggesting that nonresponse bias is not a problem in our sample.

The final sample included 67% females. The average age of the sample participants was 31 years. Respondents’ average experience was 9 years. The average tenure with the same hotel was 3.4 years. Among respondents, 49% had a bachelor’s degree, 45% had a master’s degree, and 6% had completed high school.

### Measures

All measures were retrieved from the existing literature. However, according to Chinese respondents, all measures were first translated into Chinese and then back-translated into English by four language experts [68]. This approach is validated by a large number of studies [51, 69–73]. After completing the translations, we invited two HR professionals from the sample organizations to evaluate the content of the survey measures. Based on their recommendations, we made minor modifications to the final survey. This process of translation and review by language experts and industry professionals enhanced the face and content validity of the survey measures. All measures were reported using 7 points Likert scale (1 = strongly disagree to 7 = strongly agree).

#### Humble leadership

We used a nine-item scale to measure humble leadership [3]. Sample item was “My leader is open to the advice of others.” The Cronbach’s alpha for this measure was 0.91.

### Leader-member exchange

We adopted a seven-item scale of Wayne, Shore [56] to measure leader-member exchange. Sample items were “I usually know where I stand with my manager.” and “My manager has enough confidence in me that he/she would defend and justify my decisions if I was not present to do so.” The Cronbach’s alpha for this measure was 0.97.

### Core self-evaluation

We used twelve items developed by Judge, Erez [26] to measure employee CSE. Sample items were “I complete tasks successfully,” and “Overall, I am satisfied with myself.” The Cronbach’s alpha for this measure was 0.97.

### Employee innovative behavior

We used a six-item scale to measure employee innovative behavior. This measure was developed by Scott and Bruce [19]. We asked direct supervisors of the employees to rate the innovative performance of their subordinates. This approach is validated by previous research and is considered valid compared with self-reported performance measures [20, 74]. Sample items were, this employee… “Investigates and secures funds needed to implement new ideas,” and “Develops adequate plans and schedules for the implementation of new ideas.” The Cronbach’s alpha for this measure was 0.88.

### Control variables

Previous research on leadership and innovation suggests that demographic variables influence employee behavior and performance [69, 75, 76]. Therefore, we controlled the effect of employee age, gender, education, and organizational tenure.

## Results

### Analytical strategy

A two-step approach suggested by Anderson and Gerbing [77] was used to assess the data reliability and hypotheses testing. In particular, we used AMOS to test the reliability of the data and PROCESS macro in SPSS to test the model. A set of confirmatory factor analyses using AMOS were conducted to test the reliability of the data. Then PROCESS macro was used to test the model [78].

### Descriptive statistics and confirmatory factor analysis

Table 1 shows the means, standard deviation, correlations, and Cronbach’s alpha values. We performed confirmatory factor analysis on the four-factors hypothesized model. Results exhibited good model fit indices (χ2 = 950.95, df = 511, GFI = 0.86, TLI = 0.96, CFI = 0.97, RMSEA = 0.05). We then performed a set of confirmatory factor analyses to test the alternative model to assess the discriminant validity. Results suggest that hypothesized model produced a better fit than the alternative three-factor model in which we combined humble leadership with leader-member exchange (χ2 = 4042.39, df = 514, GFI = 0.63, TLI = 0.70, CFI = 0.73, RMSEA = 0.15), two factors model in which we combined humble leadership, leader-member exchange, employee CSE (χ2 = 5303.20, df = 516, GFI = 0.40, TLI = 0.60, CFI = 0.63, RMSEA = 0.17), and single factor model in which all measures were combined (χ2 = 6085.41, df = 517, GFI = 0.41, TLI = 0.53, CFI = 0.57, RMSEA = 0.18). Thus, the alternative model tests demonstrate discriminant validity of the data.

**Table 1:** Correlation matrix

Variables	Mean	Std. Deviation	1	2	3	4	5	6	7	8
1. Gender	1.33	0.47	-							
2. Age	31.25	6.97	0.04	-						
3. Education	3.39	0.60	-0.02	0.06	-					
4. Organizatio-nal tenure	3.40	1.82	0.03	-0.04	-0.04	-				
5. humble leadership	4.17	0.79	-0.03	0.02	0.02	-0.05	0.91			
6. LMX	3.65	1.23	-0.02	-0.01	0.07	0.10	0.19**	0.97		
7. CSE	3.72	1.24	0.00	0.05	-0.08	-0.02	-0.37**	-0.03	0.97	
8. Follower innovation behavior	5.16	1.29	0.02	0.04	0.11*	-0.06	0.39**	0.20**	-0.30**	0.88

*Note*: *N* = 328, LMX = Leader-member exchange, CSE = core self-evaluation; **p ≤.01; Cronbach’s alpha values in diagonal cells

Research suggests that self-reported measures are prone to the issue of common method bias (CMB) [79]. To mitigate potential CMB issues, we initially utilized time-lagged, multi-source data in which the innovative behavior of employees was rated by their direct supervisors. This approach is recommended to help reduce the potential impact of common method bias [80]. After collecting the data, we conducted Harman’s single-factor analysis to assess whether the data were affected by CMB [81]. The results suggest that the first factor accounts for 33.69% of the total variance. Thus, these results provide further evidence that our findings are not affected by common method bias.

### Hypothesis testing

Results in Table 2 show the results of the hypotheses testing analysis. As predicted in Hypothesis 1, humble leadership is positively related to employee innovative behavior. Results show that there is a positive relationship between humble leadership and employee innovative behavior (β = 0.36, *p* <.001). Thus, Hypothesis 1 was supported.

**Table 2:** Results of regression analysis

	Follower innovation behavior		Follower innovation behavior		LMX		Follower innovation behavior	
Variables	β	t	β	t	β	t	β	t
**Controlled effects**								
Gender	0.03	0.68	0.03	0.51	-0.02	-0.36	0.07	0.63
Age	0.02	0.43	0.03	0.61	-0.02	-0.34	0.01	1.06
Education	0.09	1.73	0.10	1.92	0.07	1.36	0.10	1.11
Organizational tenure	-0.05	-1.03	-0.04	-0.86	0.11	2.02*	-0.04	-1.39
**Main effects**								
Humble leadership	0.36	6.94***	0.34	6.38***	0.19	3.54***		
LMX	0.13	2.45**					0.18	3.56****
CSE			0.24	4.31***			0.29	5.72***
**Moderating effects**								
Interaction 1			0.17	3.87***				
Interaction 2							0.14	2.68**

*Note*: *N* = 328, LMX = Leader-member exchange, CSE = core self-evaluation; Interaction 1 = humble leadership x CSE; Interaction 2 = LMX = CSE; **p ≤.01; ***p ≤.001

**Fig. 2:**
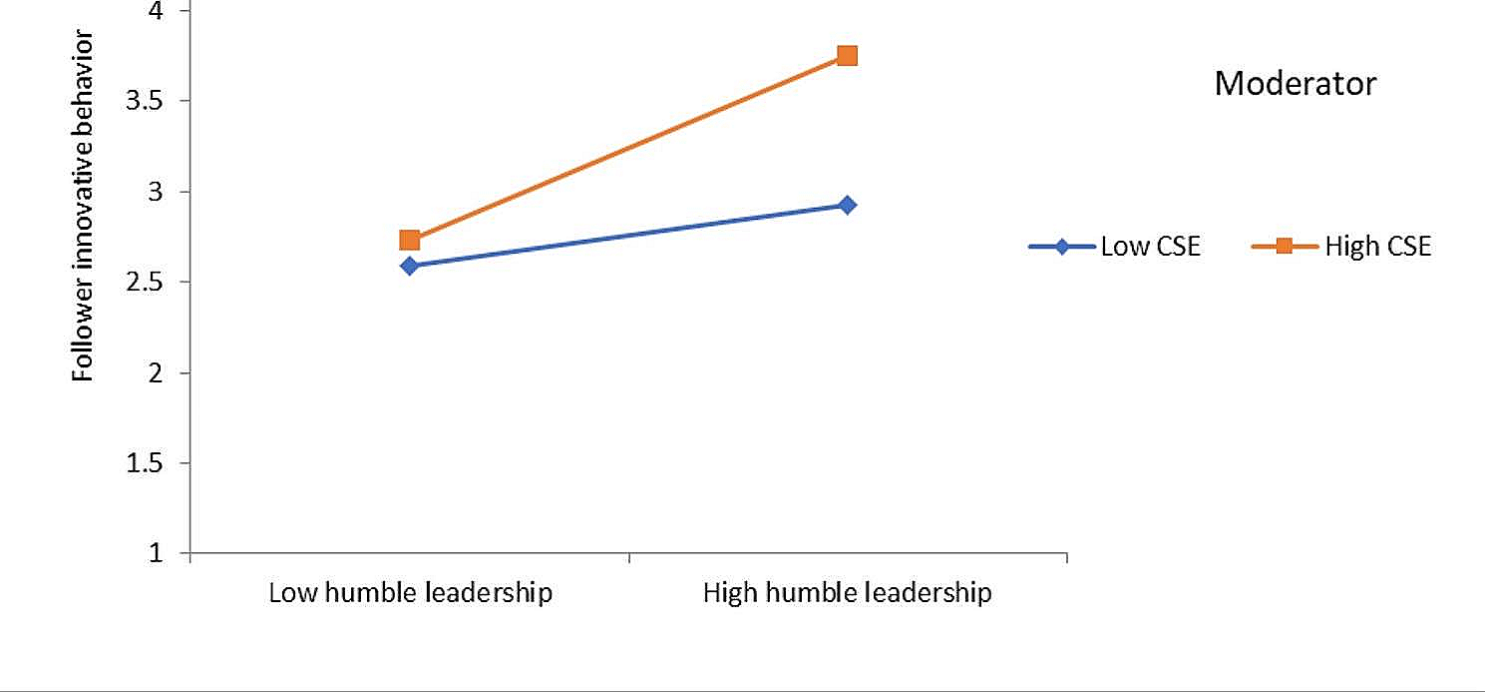
Interaction effects of spiritual leadership and CSE on employee innovative behavior

Hypothesis 2 predicted that followers’ CSE moderates the relationship between humble leadership and innovative behavior of followers, such that the relationship is stronger among followers with high rather than a low level of CSE. Results reveal that the interaction effect of humble leadership and follower CSE was significant on employee innovative behavior (β = 0.17, *p* <.001). To further understand the moderating effect, we followed Aiken and West [82] and performed a simple slope test. The finding is plotted in Fig. 2. Results of simple slope analysis reveal that when CSE is high the impact of humble leadership is strongest on employee innovative behavior (β = 0.51, *p* <.001) than at a low level of employee CSE (β = 0.17, *p* <.01). Thus, these results provide support for Hypothesis 2.

Hypothesis 3 predicted that humble leadership is positively related to LMX. Accordingly, results provide support for Hypothesis 3 and reveal that humble leadership has a positive relationship with LMX (β = 0.19, *p* <.001).

Hypothesis 4 predicted that LMX mediates the relationship between humble leadership and follower innovative behavior. Accordingly, results in Table 2 reveal that LMX mediates the positive relationship between humble leadership and follower innovative behavior (indirect effect = 0.03, SE = 0.10, 95% CI [0.01, 0.05]). Thus, the results provide support for Hypothesis 4.

**Fig. 3:**
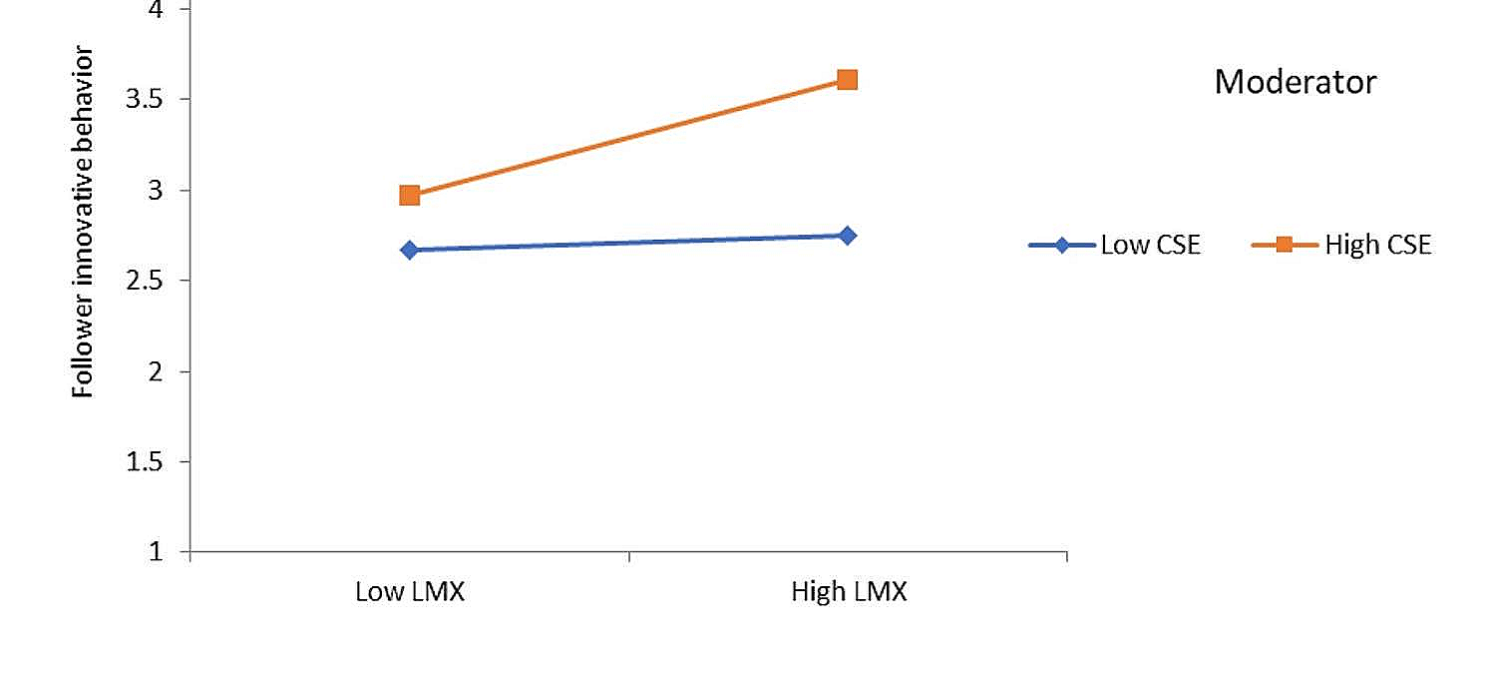
Interaction effects of LMX and CSE follower innovative behavior

Hypothesis 5 a predicted that CSE moderates the relationship between LMX and follower innovative behavior, such that the relationship between LMX and follower innovative behavior is stronger among followers with high rather than low levels of CSE. Results in Table 2 reveal that there is a significant interaction effect of CSE and LMX on follower innovation behavior (β = 0.14, *p* <.01). To further understand the moderating effect, we followed Aiken and West [82] and performed a simple slope test. The finding is plotted in Fig. 3. Results of simple slope analysis reveal that when CSE is high the impact of LMX is strongest on employee innovative behavior (β = 0.32, *p* <.001) than at a low level of employee CSE (β = 0.04, ns). Thus, these results provide support for Hypothesis 5a.

Finally, mediated moderated hypothesis of this study was tested using the three-step approach recommended Muller, Judd [83]. Results of mediated moderation Hypothesis 5b are reported in Table 3. First, after controlling the effects of control variables, we regressed humble leadership and CSE on follower innovative behavior. Results reveal a significant effect of humble leadership (β = 0.34, *p* <.001) and the interaction term (β = 0.17, *p* <.001) on follower innovative behavior. Second, after controlling the effects of control variables, we regressed humble leadership, CSE, and interaction on LMX. Results reveal that humble leadership significantly (β = 0.22, *p* <.01) and interaction insignificantly (β = 0.03, ns) influences LMX. Third, after controlling the effect of control variables, we regressed humble leadership, LMX, and interaction effects of humble leadership and CSE and LMX and CSE on follower innovative behavior. Results reported in Table 3 reveal that the effect of both interaction terms [(LMX and CSE) β = 0.12, *p* <.01; (humble leadership and CSE) β = 0.15, *p* <.001)] are significant. However, the interaction effect of humble leadership and CSE becomes weaker. The interaction effect of LMX and CSE on follower innovative behavior is plotted in Fig. 3. Overall, the results suggest that the relationship between humble leadership and follower innovative behavior was mediated by LMX, and the relationship between LMX and follower innovative behavior was strengthened by CSE. Thus, the findings support Hypothesis 5b and suggest a mediated moderation effect.

**Table 3:** Analysis of mediated moderation

	Follower innovation behavior		LMX		Follower innovation behavior	
Variables	β	t	β	t	β	t
Gender	0.05	0.51	-0.04	-0.36	0.07	0.71
Age	0.00	0.61	0.00	-0.40	0.01	0.86
Education	0.16	1.92	0.13	1.48	0.13	1.58
Organizational tenure	-0.02	-0.86	0.06	2.07*	-0.03	-1.00
Humble leadership	0.34	6.38***	0.22	3.72***	0.31	5.83***
CSE	0.24	4.31***	-0.05	-0.85	0.24	4.36***
Interaction 1	0.17	3.87***	0.03	0.64	0.15	3.44***
LMX					0.12	2.50**
Interaction 2					0.12	2.44*

*Note*: *N* = 328, LMX = Leader-member exchange, CSE = core self-evaluation; Interaction 1 = humble leadership x CSE; Interaction 2 = LMX = CSE; *p ≤.05; **p ≤.01; ***p ≤.001

## Discussion

This research aimed to examine how followers’ positive psychological resources, specifically CSE, and relational processes, such as leader-member exchange, might impact the relationship between humble leadership and follower innovative behavior within a comprehensive model that integrates mediation and moderation. Our study revealed that the association between humble leadership and follower innovative behavior is moderated by CSE. Particularly, high CSE followers show a stronger association between humble leadership and innovative behavior than low CSE followers. Additionally, we found that LMX plays a mediating role in this relationship, which is further conditional and dependent upon the adherents’ CSE. These results have significant implications for both theoretical understanding and practical applications.

### Theoretical implications

A key contribution of our study is revealing a critical conditionality for the impact of humble leadership on followers’ innovative behavior, thereby providing empirical validation and progression to the initial theoretical amalgamation of humble leadership and CSE [25, 75]. The results of this study imply that the harmonious alignment between leadership conduct and follower cognitive assets fosters follower innovativeness. To be more specific, we found that followers with a high level of CSE were expected to exhibit greater levels of innovative behavior when their CSE was augmented by a more humble leadership-oriented leadership style than when they possessed a low level of CSE.

The results of this study not only respond to the call for an integrated approach to the research on humble leadership and follower CSE [39, 63, 84] but emphasize the importance of embracing a complementary viewpoint in leadership studies [31, 38]. The conventional approach to studying leadership impact has emphasized the augmentative role of followers’ characteristics. However, the complementarity perspective introduces a fresh perspective on the effectiveness of leadership and its underlying mechanisms. It suggests that leadership effectiveness could be better understood by exploring the interplay between leaders and followers, rather than viewing them as distinct entities. In this regard, future research should not only focus on personal traits like followers’ CSE but also investigate how work tasks and other prospective organizational contexts may either complement or supplement humble leadership [85, 86]. Future research on humble leadership should include the incorporation of such contingency variables.

This study makes a theoretical and empirical contribution by exploring how the association between humble leadership and their followers’ innovative behavior is mediated by relational processes, specifically LMX. Our findings highlight the significance of embracing a relationship-centered viewpoint in (humble) leadership study and contribute to our understanding of the effectiveness of humble leadership [28, 56, 59]. The findings of this study reveal that the humble leadership and follower innovative behavior relationship is subject to moderation by CSE, and is further explained by the mediating role of LMX. Specifically, the results demonstrate that humble leadership is conducive to LMX, which in turn enhances the innovative behavior of followers who possess a high level of CSE. This research establishes a pathway through which humble leadership complements the positive psychological resources of followers, ultimately leading to their innovation. By introducing a mediated moderation model, this study highlights the importance of integrating moderators and mediators within a single theoretical framework to gain a more nuanced understanding of the intricacy of humble leadership.

The outcomes of our study strengthen the theoretical framework of alternatives for leadership. This classic but insufficiently investigated perspective on leadership suggests that certain subordinate, task and organizational features possess the capability to substitute or counterweigh the effects of leadership, resulting in a decline in a leader’s capacity to stimulate adherents’ effectiveness. As an illustration, Bauer, Erdogan [59] revealed that managers with introverted personalities require a high-quality LMX relationship to achieve optimal performance, while extroverts are capable of seeking social interaction, resources, and support independently of such a relationship. This implies that CSE induces the impact of both humble leadership and LMX, serving as a key resource for follower innovative behavior.

### Limitations and future research directions

Prior to discussing the practical implications of our study, it is important to acknowledge prospective limitations. Firstly, the longitudinal data nature of our data cannot account for causality issues. Therefore, future research is suggested to use experimental design to replicate the findings of this study. Secondly, common method bias may be a concern since humble leadership, CSE, and LMX data were obtained from similar participants (i.e., followers), which could lead to an inflated correlation between these constructs. Nevertheless, our time-lagged data in which the outcome is leader-rated can potentially mitigate the influence of common method bias [87]. Moreover, the results from the confirmatory factor analysis offer partial sustenance for the discriminant validity of humble leadership, CSE, and LMX measures. However, it is important to acknowledge that this empirical design bars us from constructing causal inferences, and the potential for common method bias exists as data on humble leadership, CSE, and LMX were obtained from the same source. To address this limitation, further research may employ a longitudinal approach and gather information through numerous sources.

It is possible that relying on the innovative behavior of followers’ ratings given by leaders could be a constraint. While these ratings can provide valuable insight into the perceptions and behaviors of leaders and followers, they are not without potential drawbacks. For example, they may be influenced by biases or personal relationships between the leader and follower, as well as by factors such as the leader’s leadership style or the follower’s job performance. Additionally, such ratings may not be reflective of broader organizational performance or objective measures of performance. As such, it is important for researchers to acknowledge and address the potential limitations associated with the use of these ratings in their studies, and to consider alternative sources of data such as objective reports.

Finally, the limitation that needs to be acknowledged is the generalizability of the results, given that the study’s sample is derived from a single industry in China. The humble dimension of leadership is entrenched in and supported by the organizational culture of this specific company, which is influenced by the larger societal and cultural milieu [88]. Consequently, the interpretation of “humility” by the participants in this study may differ from that of individuals in other countries or organizations, which could influence the relationships found. Therefore, caution must be exercised when extrapolating the results beyond the study’s specific context. To enhance the generalizability of the findings, future research should include a wider range of organizational and societal cultures.

### Practical implications

The current investigation adds value by synergistically merging two emerging and interlinked domains that have demonstrated practical relevance. Amid the contemporary workplace challenges related to competition and ethics, comprehending and implementing humble leadership assumes a crucial role for leaders and their followers [89]. Simultaneously, in the current fast-paced and tumultuous work environment, constructive psychological resources possess a considerable potential to confer an unacknowledged competitive edge to individuals, teams, and organizations [90, 91]. In particular, CSE has been established through numerous studies to be associated with favorable attitudes, behaviors, and performance. Additionally, empirical research has indicated that CSE is subject to change and is malleable. It can be enhanced through brief training interventions that have a proven causal impact on performance.

Based on the results indicating the positive influence of humble leadership on followers’ innovative behavior, organizations may consider focusing on developing their managers as humble leaders. Fry, Vitucci [89] have previously offered explicit recommendations for humble leadership development, as outlined in their publications such as Hannah, Walumbwa [92], Fry, Vitucci [89], and [93]. However, it is worth mentioning that not all followers may be equally open to the concept of humble leadership and the consequent exchange relationship that may develop as a result, and how it relates to innovative behavior. Our study reinforces this observation by indicating that followers with a high level of CSE may be more receptive to humble leadership as a means to enhance their innovative behavior, thereby providing opportunities to gain more leverage in this area.

Based on our research findings, humble leaders can achieve greater impact by directing their attention towards followers with a high level of CSE, as this can lead to complementarity congruity and improve overall innovative performance. Consequently, these high CSE followers could become the focus of development efforts, thereby alleviating the pressure on humble leaders to provide close attention to low CSE followers. One way to address the inclusion of employees with higher CSE is to include behavior measures in recruitment process by the human resources department. However, more efforts should be focused on increasing followers’ CSE, as a means to better benefit from certain leadership behavior for improving innovation. For instance, leaders are suggested to adopt motivational strategies to improve self-view of employees. While CSE may serve as a viable replacement leadership, the complementary development of both CSE and humble leadership can lead to a scenario where the achievement of goals results in positive outcomes for all parties involved, leading to effective performance. Moreover, effective leaders exhibit humble behaviors in the context of a dynamic and reciprocal exchange relationship. By prioritizing servant leadership, these leaders foster open communication and mutual exchange, which in turn positively impacts the innovative behavior of their followers.

To conclude, the present study successfully integrated the concepts of leadership (specifically humble leadership and LMX) and CSE, revealing that the relationship between humble leadership and followers’ innovative behavior is subject to the followers’ CSE. The results also demonstrated that the positive association between humble leadership and LMX, and ultimately, followers’ innovative behavior, is stronger among followers with a high level of CSE. These findings provide a more nuanced understanding of the intricacies of humble leadership and offer insights into how it can be more effectively utilized to enhance followers’ innovative behavior.

## Data Availability

The original contributions presented in the study are included in the article, further inquiries can be directed to the corresponding author.
